# Image-guided optimization of current steering in STN-DBS for Parkinson's disease

**DOI:** 10.3389/fneur.2025.1618480

**Published:** 2025-07-02

**Authors:** Atsushi Umemura, Hideki Mizuno, Mina Maki, Atsuo Masago

**Affiliations:** ^1^Department of Neurosurgery, Juntendo University, Tokyo, Japan; ^2^Department of Neurosurgery, Ookuma Hospital, Nagoya, Japan; ^3^Department of Neurology, Ookuma Hospital, Nagoya, Japan

**Keywords:** Parkinson's disease, subthalamic nucleus, deep brain stimulation, current steering, image-guided programming

## Abstract

**Objective:**

Recent advancements in multiple independent current control (MICC) technology, combined with directional leads, have improved clinical outcomes in subthalamic nucleus deep brain stimulation (STN-DBS) for Parkinson's disease (PD). However, these advancements have also increased the complexity and duration of programming. This study aimed to evaluate the clinical utility of image-guided programming (IGP) in patients with stable postoperative symptoms.

**Methods:**

Sixteen patients with advanced PD, who had undergone STN-DBS and exhibited stable symptoms for at least 6 months under conventional programming, were enrolled. An alternative stimulation program was developed using Stimview™ XT, a patient-specific image-guided interface, without altering pulse width or frequency. Stimulation fields were modified using horizontal and vertical current steering based on individual STN anatomy. Motor function was evaluated via the Unified Parkinson's Disease Rating Scale part III (UPDRS III) before, 1 h after, and 3 months post-reprogramming.

**Results:**

Image-guided current steering resulted in modifications in 29 of the 32 leads. Horizontal steering was newly introduced in 23 leads, while vertical adjustments were made in six leads. Nine patients reported immediate subjective improvement, and 15 of 16 opted to continue with the IGP-derived settings. Statistically significant improvements in Unified Parkinson's Disease Rating Scale (UPDRS) part III scores were observed 1 h after reprogramming (*P* < 0.05), and these improvements were sustained at the 3-month follow-up.

**Conclusions:**

IGP provides a clinically effective, time-efficient strategy for refining current steering in STN-DBS, even in patients with stable symptoms under conventional settings. By leveraging individualized anatomical visualization, IGP enhances precision in targeting the dorsolateral STN, resulting in improved motor outcomes.

## 1 Introduction

Deep brain stimulation (DBS) targeting the subthalamic nucleus (STN) has emerged as one of the most effective surgical interventions for patients with Parkinson's disease (PD) who are refractory to pharmacological treatment. Subthalamic nucleus deep brain stimulation (STN-DBS) not only improves motor symptoms during medication-off periods but also reduces dyskinesia through decreased reliance on dopaminergic medications. The success of STN-DBS is contingent upon three critical elements: appropriate patient selection, accurate lead placement, and optimal programming of stimulation parameters.

Surgery of STN-DBS is usually performed by combination with MRI-guided stereotactic procedure and microelectrode recording. The most important thing in STN-DBS surgery is to place the 4-contact DBS lead in the center of the STN. If the lead is not successfully placed in the center of the STN, a sufficient area of the STN is not stimulated with conventional ring electrode. Futhermore, stimulation may spread beyond the STN and induce some adverse effects. For these situations, recent new technology of the multiple independent current control (MICC) (1–3) with the directional lead (4–6) enables various current steering. While conventional ring electrode creates spherical electrical field, MICC directional lead may create much better electrical field fitting the shape of the STN (7).

Studies using imaging and electrophysiological methods have shown that the most effective stimulation locations for alleviating motor symptoms in PD vary. Reported optimal stimulation sites include the dorsolateral (8–11), lateral (11, 12), within (13), and posterolateral (14) regions of the STN, as well as the white matter dorsal to the STN (15). From these findings, the lateral and particularly dorsolateral regions of the STN are considered the most effective stimulation sites for improving motor symptoms. Therefore, programming to focusly stimulate the dorsolateral STN using current steering can provide better clinical efficacy and help avoid stimulation-induced adverse effects. However, actual programming using current steering has become more complicated in clinical situation. Adjusting stimulation with the conventional method by checking the patient's motor symptoms has become a cumbersome task that requires a lot of experience and time.

To date, image-guided programming (IGP) has been utilized to facilitate efficient programming in STN-DBS for PD, demonstrating clinical outcomes comparable to those achieved with conventional programming methods (16–21). Recently, new software integrated into the stimulation programmer has been developed to support IGP specifically for systems employing MICC directional leads. This new feature of the stimulation programmer displays the STN and surrounding structures in relation to the DBS lead in 3D for each patient from preoperative MRI images and CT images after lead placement. Moreover, it allows for the objective design of stimulation programs by rendering the resulting stimulation field using current steering, thereby enabling more precise and effective targeting of the STN.

The present study aimed to evaluate the clinical utility of IGP in patients who had undergone STN-DBS using MICC directional leads and were in the chronic postoperative phase with stabilized motor symptoms. Specifically, we investigated whether the application of IGP could further optimize stimulation fields and enhance motor outcomes in this cohort.

## 2 Methods

### 2.1 Participants

This study enrolled 16 advanced PD patients who underwent STN-DBS with MICC directional lead system (Boston Scientific, Valencia, CA, USA) for motor complication of levodopa and were followed up for more than 6 months after surgery [nine men and seven women, age 68.3 ± 1.8 years, mean ± standard deviation (SD)]. All patients did not exhibit show any significant motor fluctuations and dyskinesias after STN-DBS. They were generally satisfied with the surgical outcomes. In this study, we applied a program created under image guidance to these patients whose symptoms had stabilized with conventional programs, and observed changes in subjective and objective motor symptoms.

All participants provided written informed consent. This study was approved by our Institutional Review Board (approved number: 23-07).

### 2.2 Lead placement procedure

Directional leads (Vercise CasteriaTM: Boston Scientific, Valencia, CA, USA) were implanted into the bilateral STN stereotactically with MR imaging guidance under local anesthesia. The target localization was based on direct visualization of the MR image using surgical planning software (Frame Link, Medtronic). The target was refined physiologically by intraoperative single tract microelectrode recording. Details of surgical procedure were described previously (22). When a sufficient length of STN activity was obtained (length more than 4.5 mm), we considered the trajectory to have passed through almost the center of the STN and implanted the DBS lead at the trajectory. If sufficient STN activity was not obtained, altered trajectories were followed until sufficient STN activity was obtained. The lead was placed as the most distant contact (contact 1) of the lead placed at the bottom of the STN. In this situation, two middle contacts (directional segment) are placed at the center and the upper portion of the STN. Radio-dense marker is equipped to know the direction of the lead. The directional lead should be placed as the marker faces forward. However, this orientation was not always accurate.

### 2.3 Initial manual programming

DBS programming usually began 7 days after surgery with conventional method by checking the patient's symptoms. The programming method was as follows. Firstly, we determine the most effective contact level for cardinal PD symptoms by monopolar review using ring mode stimulation. We usually evaluate therapeutic window in all four contact levels and choose the contact with the widest therapeutic window. In our method of lead implantation, either of two middle contact level (3-segmented contact level) is usually chosen as the most effective contact level. Stimulation is begun by ring-mode with stimulation parameters, 60 μs of pulse width, 130 Hz of pulse rate, and 0.5 mA of amplitude. Then, amplitude is gradually increased, and dopaminergic medication is gradually reduced based on stimulation induced improvements of PD symptoms.

After initial programming, current steering is evaluated to explore more effective stimulation setting. Vertical current steering is achieved by additional activation of next level contact. Vertical steering creates wider stimulation field and may produce better effects. In some patients, additional activation of upper-level contact improves dyskinesia or tremor by stimulation of the dorsal area to the STN. Horizontal current steering is also achieved by changing current amplitude distribution in 3-segmented contacts. Most effective distribution is selected depending on symptom relief. If some kinds of stimulation-induced adverse effects such as spasticity, dysarthria, paresthesia emerge, and drastic horizontal steering (e.g., use of only one segmented contact) is employed to avoid it.

After these process, optimization of the stimulation setting is continued periodically in out-patient clinic. However, conventional programming using current steering is actually very complex and time consuming.

### 2.4 Image guided programming with Stimview™ XT

We systematically reprogrammed the stimulation setting by IGP in the chronic stage after surgery using the commercially available image-guided programming software, Stimview™ XT (Boston Scientific, Valencia, CA, USA). This software is built into the stimulation control programmer for Boston Scientific DBS. To start Stimview™ XT, the preoperative thin-slice T1- and T2-weighted MR images and the most recent CT images showing DBS leads were prepared. CT slices should be taken perpendicular to the lead as much as possible. First, these images were fused on Stimview™ XT. Then, anatomical mapping was performed with MR images. We especially displayed images of the STN, red nucleus (RN), and substantia nigra (SN). Finally, lead localization was performed to identify the lead orientation. This lead localization feature of the Stimview™ XT assesses the CT artifacts of radiopaquemarker of the directional lead to identify the precise direction of the lead automatically. These preparations are made for each patient.

Usually, programming with Stimview™ XT is done while examining the patient face-to-face. Once the Vercise neural navigator is launched and connected to the patient's implantable pulse generator, the patient's unique anatomy of the STN, RN, and SN is visualized in addition to the orientated leads and the volume of tissue activate (VTA). Then, while observing the patient's symptoms, current steering is used to create a stimulation field that matches the shape of the STN.

Another option is to use Stimview™ XT to create optimal programming in advance and then apply it directly to patients in the outpatient clinic. Demo mode of the Vercise neural navigator is launced. First, display the positional relationship between the STN and the lead whose orientation has been identified. Next, input the current program and display the VTA. After that, modify the VTA so that it fits the shape of the STN by using current steering. Practically, horizontal and vertical current steering was used to create a stimulation field that stimulates the dorsolateral STN surely without the stimulation spreading outside of the STN. The 3D split view of Stimview™ XT was particularly useful in creating an appropriate program. We used this method to reprogram DBS setting in each patient. In principle, the stimulation amplitude, pulth width and frequency were not changed in this study. This pre-programming method is believed to be more time-saving than face-to-face programming. Actually, the time required to create a image-guided programming was ~15 min in each patients.

### 2.5 Assessment of motor function

We applied a new image-guided program to all participants in out-patient clinic. Then, we interviewed participants about their subjective evaluation of the program changes. If the participants did not exhibit like the changed program, they were allowed to revert to the original settings at any time.

We also investigated the Unified Parkinson's Disease Rating Scale (UPDRS) III motor scores before and 1-h after changing to image-guided program. Assessment of Unified Parkinson's Disease Rating Scale part III (UPDRS III) motor score was also performed 3 months after changing to image-guided program in out-patient clinic. The dose of dopaminergic medication was not changed during this period to confirm the effect of the programming change.

### 2.6 Statistical analysis

Differences in UPDRS Part III motor scores before and after reprogramming were analyzed using two-tailed paired *t*-tests. To account for multiple comparisons across different time points (baseline, 1 h post-programming, and 3 months post-programming), Bonferroni correction was applied to control for family-wise error rate. Corrected *P*-values < 0.05 were considered statistically significant. All statistical analyses were performed using Microsoft Excel.

## 3 Results

### 3.1 Changes in current steering by image-guided programming

The clinical data of the participants are shown in Table 1. The stimulation field was modified using current steering by image-guided program in 29 of the 32 leads in total. In three leads (Case 8 left, Case 9 left, Case 11 left), the stimulation field did not exhibit change from that of the conventional manual program even with the image-guided program.

**Table 1:** Clinical data of the participants.

**Case no**.	**Age/sex**	**Postoperive period (months)**	**Initial program contact allocation, Amp (mA), PW (μs), Fre (Hz), Pattern of current steering**	**Image-guided program (IPG) Contact allocation, Amp (mA), PW (μs), Fre (Hz), Pattern of current steering**	**UPDRSIII before IPG**	**UPDRSIII after IGP**	**UPDRSIII 3 months after IGP**	**Participant self-assessment**
1	74/F	51	Lt: 5 (30%) 6 (30%) 7 (30%) 8 (10%), 3.5, 60, 130, Vertical Rt: 2 (34%) 3 (33%) 4 (33%), 2.9, 60, 130, Ring	Lt: 5 (68%) 7 (22%) 8 (10%), 3.5, 60, 130, Vertical and Horizontal Rt: 2 (18%) 3 (16%) 4 (16%) 5 (18%) 6 (16%) 7 (16%), 2.9, 60, 130, Vertical	30	25	22	No change
2	66/M	49	Lt: 5 (34%) 6 (33%) 7 (33%), 2.1, 60, 130, Ring Rt: 2 (10%) 3 (10%) 4 (10%) 5 (24%) 6 (23%) 7 (23%), 3.8, 60, 130, Vertical	Lt: 5 (25%) 7 (75%), 2.1, 60, 130, Horizontal Rt: 4 (20%) 7 (80%), 3.8, 60, 130, Vertical and Horizontal	18	14	14	Improved mood
3	64/M	43	Lt: 5 (34%) 6 (33%) 7 (33%), 2.7, 60, 130, Ring Rt: 5 (34%) 6 (33%) 7 (33%), 2.4, 60, 130, Ring	Lt: 2 (8%) 3 (6%) 4 (6%) 5 (28%) 6 (26%) 7 (26%), 2.7, 60, 130, Vertical Rt: 6 (25%) 7 (75%), 2.4, 60, 130, Horizontal	15	11	12	Improved walking
4	75/M	39	Lt: 2 (20%) 3 (20%) 4 (20%) 5 (14%) 6 (13%) 7 (13%), 2.0, 60, 130, Vertical Rt: 2 (8%) 3 (6%) 4 (6%) 5 (28%) 6 (26%) 7 (26%), 2.7, 50, 130, Vertical	Lt: 4 (80%) 7 (20%), 2.0, 60, 130, Vertical and Horizontal Rt: 2 (15%) 4 (5%) 5 (60%) 7 (20%), 2.7, 50, 130, Vertical and Horizontal	28	28	30	No change
5	68/F	35	Lt: 5 (34%) 6 (33%) 7 (33%), 2.7, 60, 130, Ring Rt: 5 (34%) 6 (33%) 7 (33%), 3.0, 60, 130, Ring	Lt: 5 (50%) 6 (50%), 2.8, 60, 130, Horizontal Rt: 2 (8%) 4 (22%) 5 (18%) 7 (52%), 3.0, 60, 130, Vertical and Horizontal	22	15	15	Improved walking
6	69/F	31	Lt: 2 (34%) 3 (33%) 4 (33%), 3.2, 60, 130, Ring Rt: 5 (34%) 6 (33%) 7 (33%), 3.4, 60, 130, Ring	Lt: 3 (50%) 4 (50%), 3.2, 60, 130, Horizontal Rt: 3 (25%) 4 (25%) 6 (25%) 7 (25%), 3.4, 60, 130, Vertical and Horizontal	12	8	9	Improved walking
7	56/M	30	Lt: 5 (34%) 6 (33%) 7 (33%), 2.9, 60, 179, Ring Rt: 7 (100%), 3.6, 90, 179, Horizontal	Lt: 7 (75%) 6 (25%), 2.9, 60, 179, Horizontal Rt: 5 (25%) 7 (75%), 3.6, 90, 179, Horizontal	13	9	4	Improved mobility
8	81/F	29	Lt: 5 (34%) 6 (33%) 7 (33%), 2.0, 60, 130, Ring Rt: 2 (34%) 3 (33%) 4 (33%), 3.0, 60, 130, Ring	Lt: 5 (34%) 6 (33%) 7 (33%), 2.0, 60, 130, Ring Rt: 2 (25%) 4 (75%), 3.0, 60, 130, Horizontal	10	5	8	No change
9	64/M	29	Lt: 5 (20%) 6 (20%) 7 (20%) 8 (40%), 2.5, 60, 130, Vertical Rt: 5 (20%) 6 (20%) 7 (20%) 8 (40%), 3.4, 60, 130, Vertical	Lt: 5 (20%) 6 (20%) 7 (20%) 8 (40%), 2.5, 60, 130, Vertical Rt: 5 (45%) 7 (15%) 8 (40%), 3.4, 60, 130, Vertical and Horizontal	14	10	8	Improved freezing of gait
10	70/M	27	Lt: 2 (34%) 3 (33%) 4 (33%), 3.1, 60, 130, Ring Rt: 2 (34%) 3 (33%) 4 (33%), 3.3, 60, 130, Ring	Lt: 2 (75%) 3 (25%), 3.1, 60, 130, Horizontal Rt: 2 (75%) 3 (25%), 3.3, 60, 130, Horizontal	21	13	13	Improved right side mobility
11	77/F	26	Lt: 2 (8%) 3 (6%) 4 (6%) 5 (28%) 6 (26%) 7 (26%), 2.5, 60, 130, Vertical Rt: 2 (40%) 5 (60%), 3.8, 60, 130, Vertical and Horizontal	Lt: 2 (8%) 3 (6%) 4 (6%) 5 (28%) 6 (26%) 7 (26%), 2.5, 60, 130, Vertical Rt: 2 (30%) 4 (10%) 5 (45%) 7 (15%), 3.8, 60, 130, Vertical and Horizontal	22	19	19	No change
12	52/F	25	Lt: 5 (34%) 6 (33%) 7 (33%), 2.5, 60, 130, Ring Rt: 5 (34%) 6 (33%) 7 (33%), 2.8, 60, 130, Ring	Lt: 7 (100%), 2.5, 60, 130, Horizontal Rt: 6 (100%), 2.8, 60, 130, Horizontal	16	11	9	No change
13	71/F	18	Lt: 5 (34%) 6 (33%) 7 (33%), 2.8, 60, 130, Ring Rt: 5 (34%) 6 (33%) 7 (33%), 2.9, 60, 130, Ring	Lt: 6 (25%) 7 (75%), 2.8, 60, 130, Horizontal Rt: 2 (4%) 3 (3%) 4 (3%) 5 (30%) 6 (30%) 7 (30%), 2.9, 60, 130, Vertical	22	19	20	Worsening dyskinesia Revert to original program
14	69/M	17	Lt: 2 (18%) 3 (16%) 4 (16%) 5 (18%) 6 (16%) 7 (16%), 2.0, 60, 130, Vertical Rt: 5 (34%) 6 (33%) 7 (33%), 3.5, 60, 130, Ring	Lt: 2 (15%) 4 (45%) 5 (10%) 7 (30%), 2.0, 60, 130, Vertical and Horizontal Rt: 2 (8%) 3 (6%) 4 (6%) 5 (28%) 6 (26%) 7 (26%), 3.5, 60, 130, Vertical	16	10	11	No change
15	70/M	9	Lt: 5 (34%) 6 (33%) 7 (33%), 2.5, 60, 130, Ring Rt: 5 (34%) 6 (33%) 7 (33%), 3.0, 60, 130, Ring	Lt: 5 (100%), 2.5, 60, 130, Horizontal Rt: 5 (25%) 7 (75%), 3.0, 60, 130, Horizontal	14	6	4	Improved mobility and rigidity
16	66/M	6	Lt: 2 (34%) 3 (33%) 4 (33%), 2.6, 60, 130, Ring Rt: 5 (34%) 6 (33%) 7 (33%), 2.9, 60, 130, Ring	Lt: 2 (75%) 4 (25%), 2.6, 60, 130, Horizontal Rt: 2 (15%) 4 (5%) 5 (60%) 7 (20%), 2.9, 60, 130, Vertical and Horizontal	21	10	8	Overall improvement of PD symptoms

Amp; amplitude, PW; pulse width, Fre; frequency.

**Figure 1:**
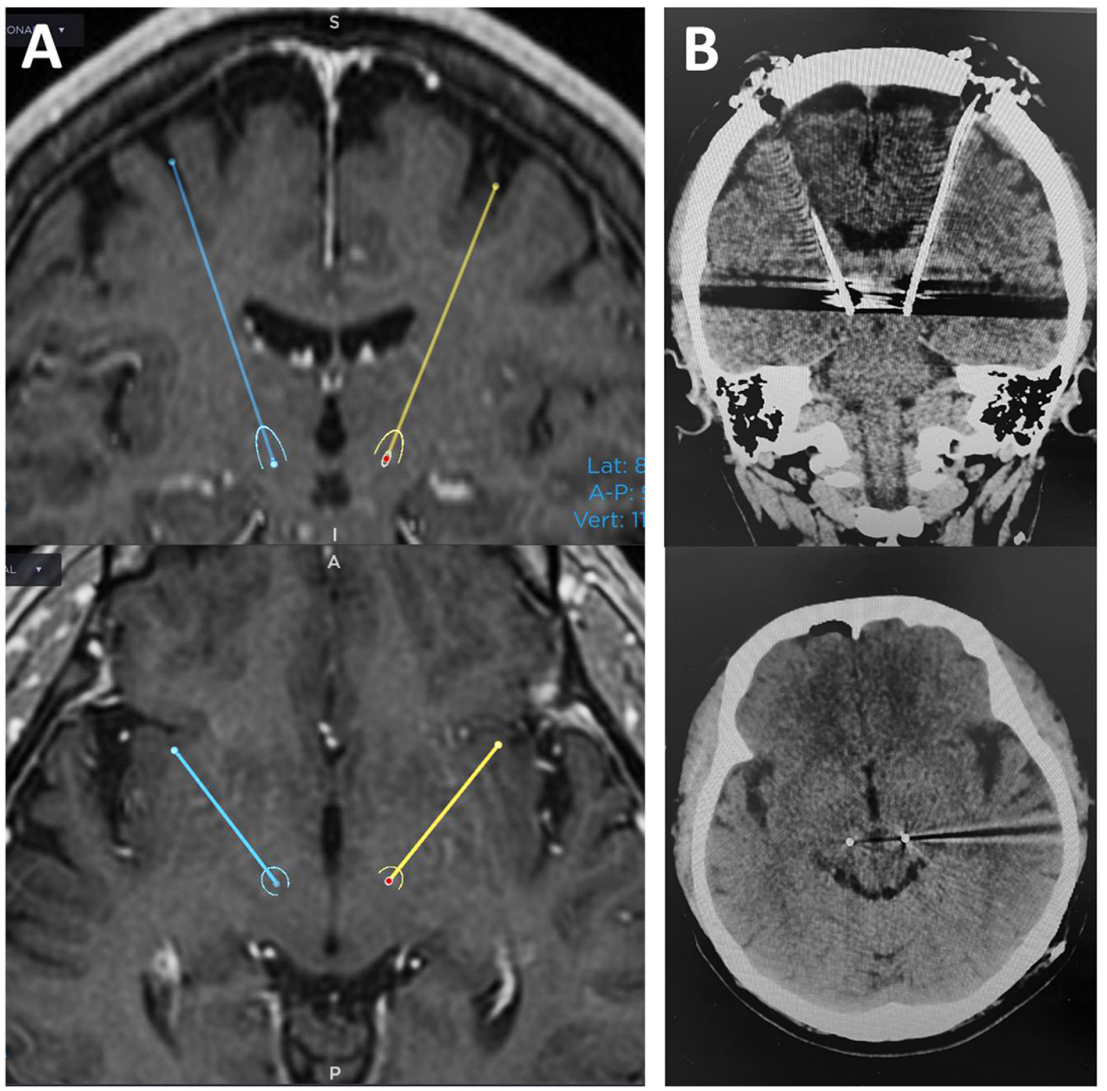
Preoperative planning and postoperative CT images in case 5. In this case, the target point (the ventral border of the STN) was 11 mm lateral to the midline, 2.5 mm posterior to the midpoint of the AC-PC, and 5 mm below the AC-PC line on both sides **(A)**. Postoperative CT images show the lead was placed almost as planned **(B)**.

**Figure 2:**
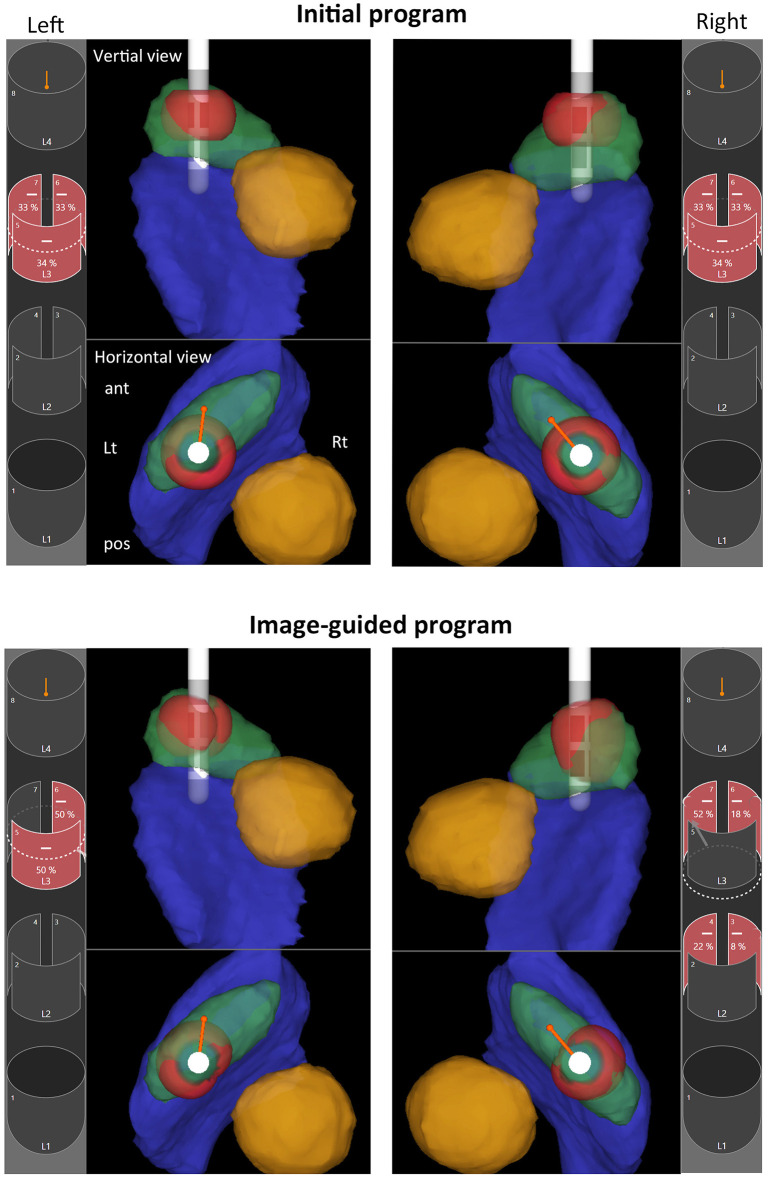
Image-guided programming performed with Stimview™ XT in case 5. A 68-year-old woman, 35 months postoperatively, had experienced a favorable clinical course without postoperative motor fluctuation or dyskinesia. However, she recently reported mild gait disturbances. Initially, the stimulation settings were configured in ring mode at the L2 level on both sides. Analysis using Stimview revealed that the left lead was placed slightly medially, with stimulation extending medially beyond the STN **(upper left)**. On the other hand, the right lead was also slightly medial, with stimulation mainly affecting the upper part of the STN **(upper right)**. Using image-guided programming, current steering was designed to ensure sufficient stimulation of the dorsolateral region of the STN. On the left side, horizontal steering was applied to shift stimulation laterally, targeting the outer portion of the STN **(lower left)**. Similarly, on the right side, horizontal steering was used to direct stimulation more laterally within the STN, and vertical steering was also applied to extend stimulation toward the central portion of the STN **(lower right)**. The patient noticed immediate improvement in gait following the stimulation adjustments. In the 3D-rendered images, anatomical structures are color-coded as follows: STN (green), red nucleus (yellow), substantia nigra (blue), and volume of tissue activated (VTA; red).

Modified patterns from the conventional manual program are summarized in Table 2. Horizontal steering was newly adopted in 23 of the 32 leads, and vertical steering was newly adopted in six of the 32 leads. Most commonly, a simple ring mode stimulation was modified to fit the shape of the STN using horizontal steering by image-guided programming in 14 leads. Pre- and postoperative images of a representative case and images of actual IGP process are shown in Figures 1, 2.

**Table 2:** Changes in current steering patterns by IGP.

**Initial program**	**Image-guided program**	**Number of leads**
Ring	Ring (no change)	1
Ring	Vertical steering	4
Ring	Horizontal steering	14
Ring	Vertical and Horizontal steering	3
Vertical steering	Vertical steering (no change)	2
Vertical steering	Vertical and Horizontal steering	6
Horizontal steering	Horizontal steering	1
Vertical and Horizontal steering	Vertical and Horizontal steering	1

### 3.2 Subjective participants self-assessment

Seven of the 16 patients did not exhibit notice any change in their symptoms even after changing to image-guided program, and were sent home with the changed program. No patient reported a worsening of symptoms immediately after the program change. However, one patient (Case 13) subsequently visited the hospital 1 month later complaining of worsening dyskinesia and requested to return to the original program.

On the other hand, nine out of 16 patients noticed some improvement in their symptoms immediately after changing to image-guided program and preferred the new program. Four patients (Case 3, 5, 6, 9) reported enhanced gait, four (Case 7, 10, 15, 16) reported reduced rigidity and improved limb mobility, and one (Case 2) reported mood improvement.

As a result, 15 of 16 patients chose to continue using the program created under image guidance even after 3 months.

### 3.3 Objective motor outcomes

Application of image-guided stimulation settings resulted in a statistically significant improvement in UPDRS Part III motor scores. The mean baseline score was 18.4 ± 5.7, which improved to 13.3 ± 6.5 1 h after reprogramming (*P* < 0.05, Bonferroni-corrected). This improvement was sustained at the 3-month follow-up, with a mean score of 12.9 ± 7.0 (*P* < 0.05, Bonferroni-corrected). These findings indicate that image-guided current steering can yield both immediate and lasting enhancements in motor function, even in patients who were previously stable under conventional programming.

## 4 Discussion

The findings of this study demonstrate that IGP using Stimview™ XT provides a practical and efficient method for optimizing stimulation parameters in patients with PD who have undergone STN-DBS with MICC directional leads. By enabling direct visualization of patient-specific anatomical structures and electrode positioning, IGP facilitates more precise current steering, which is critical for maximizing therapeutic benefit while minimizing stimulation-induced adverse effects.

Our results, showing improvements in both subjective symptomatology and objective motor function after reprogramming, align with previous studies. Torres et al. (21) reported a 21.9% improvement in the MDS-UPDRS III motor scores with IGP using GUIDE™ XT in patients who were previously refractory to conventional programming. Moreover, significant enhancements in quality of life metrics such as the PDQ-8 and EQ-VAS were noted, along with a meaningful reduction in levodopa equivalent daily doses (LEDD) in a substantial subset of patients. Rolland et al. demonstrated in a cohort of 32 patients that IGP using GUIDE™ XT led to a high concordance (78%) with expert clinical programming, and more importantly, facilitated meaningful clinical refinements in 64% of patients requiring post-operative adjustments. These refinements, primarily achieved through directional stimulation (93%), underline the unique strength of IGP in spatially targeting the dorsolateral subthalamic nucleus (STN), which is thought to correspond to the optimal stimulation site for motor symptom control. Notably, the authors reported a significant reduction in levodopa equivalent daily dose (LEDD) 1 year postoperatively, suggesting that precise stimulation might also reduce medication burden over time (20). Similarly, Waldthaler et al. (17) reported non-inferior motor outcomes between IGP and clinical programming in a prospective comparison of 29 PD patients with bilateral directional STN-DBS leads.

Notably, our study differed in that it applied IGP not exclusively to suboptimally managed patients but to a cohort with stable motor symptoms under conventional settings. Despite this, nine of 16 patients reported noticeable improvements immediately post-reprogramming, and 15 of 16 patients chose to maintain the IGP settings 3 months later, emphasizing its clinical relevance even in stable cases. This supports the hypothesis that conventional programming, although effective, may not fully exploit the spatial and physiological potential of directional leads, which can be more systematically accessed through imaging-guided approaches. One patient complained of worsening dyskinesia after IGP. The worsening of dyskinesia is thought to be a result of the enhanced stimulation effect due to optimization of the stimulation field.

One of the key advantages of IGP is its efficiency in clinical practice. Traditional DBS programming often requires extensive trial-and-error adjustments based on clinical assessments, which can be both labor-intensive and highly dependent on the experience of the clinician. In contrast, IGP allows for a more streamlined approach, reducing the time required for optimization. Our experience suggests that pre-programming stimulation settings using IGP before a clinic visit can further improve efficiency while maintaining a high level of precision. The observed improvement in UPDRS III scores after a single IGP session suggests that this method can refine stimulation fields to better align with the dorsolateral STN, the recognized “sweet spot” for motor symptom control, without increasing programming complexity or duration. Our findings are in line with Lange et al., who demonstrated that imaging-based programming significantly reduced programming time while achieving symptom control comparable to standard clinical methods.

Taken together, these studies corroborate our findings that IGP is not only feasible but also clinically effective in both suboptimally managed and stable PD patients. It provides a reproducible, anatomy-based framework that is particularly beneficial in centers with variable levels of programming expertise. While minor refinements may be required in some cases, the overall consistency and efficiency offered by IGP argue for its routine use, especially as the complexity of DBS systems continues to grow. Nonetheless, the integration of IGP should not replace clinical acumen but rather complement it. As seen in our study and those of others, patient-reported outcomes and clinician experience remain essential components in fine-tuning stimulation settings, particularly in cases where the anatomical boundaries of the STN are ambiguous or lead placement is suboptimal.

One important consideration for the broader clinical adoption of image-guided programming (IGP) is its integration into routine workflows across centers with varying levels of expertise and resources. Our experience suggests that IGP is particularly well-suited for implementation in outpatient clinics, where time and staffing limitations can constrain conventional trial-and-error programming. The ability to pre-visualize patient-specific STN anatomy and simulate stimulation fields in advance allows clinicians to prepare individualized programs efficiently, often within 15 min per patient. This approach reduces reliance on extensive in-person trial sessions and minimizes patient fatigue.

In resource-limited or less-experienced settings, IGP can serve as a decision-support tool, enhancing the confidence and consistency of stimulation parameter selection even among less seasoned programmers. It also facilitates knowledge transfer by providing a visual anatomical framework that can be shared among multidisciplinary teams. Moreover, as DBS systems grow more complex, the standardized visualization and reproducibility offered by IGP may help to harmonize programming strategies across institutions. Taken together, these advantages suggest that integrating IGP into routine DBS management not only improves clinical outcomes but also enhances workflow efficiency and accessibility across diverse healthcare environments.

## 5 Limitations

Despite the promising results, this study has certain limitations. The sample size was relatively small, and the follow-up period was limited to 3 months. Longer-term studies are necessary to assess the durability of improvements associated with IGP.

In addition, the study did not include a control group programmed with conventional methods in a blinded fashion, which may introduce bias in the subjective and objective evaluations. Although improvements in UPDRS III scores were statistically significant, the potential influence of placebo effects or patient expectations cannot be fully excluded.

Furthermore, this study did not incorporate quantitative measures of programming efficiency, such as time-to-optimal settings or clinician workload. While the image-guided approach appeared time-efficient anecdotally, future research should assess this systematically.

Finally, the anatomical accuracy of image fusion and lead localization, particularly the fidelity of the dorsolateral STN representation, may vary across individuals due to imaging resolution or registration errors. Future studies incorporating tractography or electrophysiological validation may further enhance the precision and clinical impact of IGP.

## 6 Conclusions

This study demonstrates that IGP using the Stimview™ XT platform offers a feasible and clinically effective approach for optimizing current steering in patients with Parkinson's disease undergoing STN-DBS with MICC directional leads. By leveraging patient-specific anatomical visualization and directional stimulation, IGP enables more precise targeting of the dorsolateral STN, which is associated with improved motor outcomes. Notably, even in patients with stable motor symptoms under conventional programming, IGP yielded significant subjective and objective improvements, suggesting its value as an adjunctive tool for chronic phase management.

Given its efficiency and reproducibility, IGP holds promise for standardizing DBS programming across centers with varying levels of clinical expertise. Future large-scale, controlled studies are warranted to validate these findings and assess long-term outcomes. As DBS technology continues to evolve, image-guided approaches may play an increasingly central role in enhancing therapeutic precision and optimizing patient outcomes.

## Data Availability

The original contributions presented in the study are included in the article, further inquiries can be directed to the corresponding author.
